# Brief reviews

**DOI:** 10.1590/S1980-57642012DN06020007

**Published:** 2012

**Authors:** Sonia Maria Dozzi Brucki

## THE NEUROPSYCHOLOGICAL PROFILE OF ALZHEIMER DISEASE

**Weintraub et al.** Cold Spring Harb Perspect Med 2012 doi:10.1101/cshperspect.a006171.

Dr. Weintraub et al. has written a comprehensive review of cognitive profiles among aging and dementias, comparing them to the neuropsychological deficits seen in Alzheimer's disease (AD). The authors described the neuropsychological findings regarding episodic memory, language and semantic knowledge, executive functions, working memory and attention; and visuospatial abilities. The article highlighted that although memory impairment is a hallmark of AD, it may also occur in other neurodegenerative dementias. This represents an excellent review of topics that centers on only neuropsychological findings, providing a rapid overview of underlying pathophysiological mechanisms.

## STAGING AND NATURAL HISTORY OF CEREBROVASCULAR PATHOLOGY IN DEMENTIA

**Deramecourt et al.** Neurology 2012;78:1043-1050.

Vascular cognitive impairment is a relatively new concept that encompasses mild cognitive impairment without dementia to vascular dementia and mixed dementia. The condition is a clinical diagnosis. There are presently no consensual pathologic criteria for the various degrees of cognitive decline. The authors analyzed 135 brains with varying degrees of cerebrovascular lesions (CVL) and Deramecourt et al. attempted to conceptualize the natural history of CVL. The most common lesions were vessel wall modifications (arteriolosclerosis, amyloid angiopathy, or both), followed by perivascular modifications (hemosiderin leakage, perivascular space dilatation), myelin loss and infarcts (microinfarcts and large infarcts). These findings were present in all cerebral regions. After descriptions, authors proposed a global vascular score. Brains previously considered with vascular or mixed dementia had a vascular score ≥ 10 in 98.9% whereas those considered with pure degenerative dementia had a score <10 in 92.5%.

## FRONTOTEMPORAL DEMENTIA IN ELDERLY INDIVIDUALS

**Baborie et al.** Arch Neurol 2012. Doi: 10.1001/archneurol.2011.3323.

Frontotemporal lobar degeneration (FTLD) has been considered a presenile dementia, being the second leading cause after Alzheimer's disease during this period. This manuscript shows differences in patients diagnosed with FTLD dividing them into elderly and presenile-onset FTLD. This was a retrospective study over a 25-year period (1974-2004) with 11 cases of FTLD in elderly patients and 19 cases of presenile-onset FTLD, all of which were neuropathologically identified. All elderly FTLD patients except for one had behavioral changes. Memory loss was one the presenting symptoms in 7 out of 11 the elderly patients. Subsequently, a severe degree of memory loss was present in 10 out of the 11 patients. Atrophy of temporal and frontal lobes was present in all elderly patients with FTLD but proved less severe than in patients with presenile-onset FTLD. Temporal lobe atrophy was significantly less in elderly patients in whom parietal and occipital atrophy predominated. Nine out of the 11 elderly patients with FTLD had selective loss of pyramidal neurons in the hippocampus; characteristic of hippocampal sclerosis (82%) while 37% of presenile-onset had hippocampal sclerosis. This study provides some pointers for diagnosing possibly under-recognized elderly FTLD cases.

## PRESERVATION OF NEURONS OF THE NUCLEUS BASALIS IN SUBCORTICAL ISCHEMIC VASCULAR DISEASE

**Jung et al.** Arch Neurol 2012 doi: 10.1001/archneurol.2011.2874.

The role of cholinesterase inhibitors in vascular dementia (VaD) treatment is unclear, due to heterogeneity of lesions among patients in clinical trials. Jung et al. hypothesized that in subcortical VaD, white matter lesions could lead to retrograde degeneration of the nucleus basalis (NB) neurons. Patients with subcortical ischemic vascular disease (SIVD)(n=16), AD (n=20), and mixed AD and SIVD (n=10), plus healthy controls matched for age and educational level, were compared regarding number of neurons in the NB. Loss of neurons was observed in AD and mixed groups, but not in SIVD and healthy groups. There was preservation of NB neurons in SIVD patients compared to controls. A negative correlation was observed between NB neurons and CDR scores in the AD group. These findings pointed to an absence of primary loss of cholinergic neurons in SIVD patients.

## INCREASED CEREBRAL METABOLISM AFTER 1 YEAR OF DEEP BRAIN STIMULATION IN ALZHEIMER DISEASE

**Smith et al.** Arch Neurol 2012 doi: 10.1001/archneurol.2012.590.

Last year a phase I study was published by the same group using deep brain stimulation (DBS) on fornix in six AD patients with encouraging results. In this online published study, the group investigated changes in cerebral glucose metabolism (PET) and clinical outcomes in 5 mild AD patients. After one year of DBS, two networks showed increased metabolism: a frontal-temporal-parietal-striatal-thalamic network and a frontal-temporal-parietal-occipital-hippocampal network. In similar cortical areas, higher metabolism prior to DBS was correlated with better outcomes while increased metabolism after 1 year of DBS showed same correlation with cognitive and functional measures.

## THE RELATIONSHIP BETWEEN VISCERAL ADIPOSITY AND COGNITIVE PERFORMANCE IN OLDER ADULTS

**Yoon et al.** Age Ageing 2012. Doi: 10.1093/ageing/afs018.

High body mass index (BMI) is regarded by some studies as a risk factor for cognitive decline and dementia. Yoon et al. measured abdominal adiposity by computed tomography (visceral and subcutaneous adipose tissue) and cognitive function with the Mini-Mental State Examination, in addition to BMI and waist circumference in 188 subjects <70 years of age and 62 subjects aged 70 years or older. On the multivariate logistic regression analyses, obesity (BMI >25 kg/m^2^) – odds ratio 2.61, and classification in the top tertile of visceral adiposity area – odds ratio 2.58, were associated with poor cognitive performance in subjects younger than 70 years. Adiposity is related to many mechanisms that link it to brain function.

